# Uterine fibroids and risk of preterm birth by clinical subtypes: a prospective cohort study

**DOI:** 10.1186/s12884-021-03968-2

**Published:** 2021-08-17

**Authors:** Alexandra C. Sundermann, Tiara D. Aldridge, Katherine E. Hartmann, Sarah H. Jones, Eric S. Torstenson, Digna R. Velez Edwards

**Affiliations:** 1 Vanderbilt Epidemiology Center, Institute for Medicine and Public Health, Vanderbilt University Medical Center, 2525 West End Avenue, Nashville, TN 37203 USA; 2 Women’s Health Research at Vanderbilt, Vanderbilt University Medical Center, 2525 West End Avenue, Nashville, TN 37203 USA; 3 Division of Quantitative Sciences, Department of Obstetrics and Gynecology, Vanderbilt University Medical Center, 1211 Medical Center Drive, Nashville, TN 37212 USA

**Keywords:** Uterine Fibroids, Leiomyoma, Premature Birth, Preterm Labor, Preterm Premature Rupture of Membranes, Ultrasound

## Abstract

**Background:**

Fibroids are present in approximately one in ten pregnancies and are inconsistently linked with preterm birth. We sought to determine the association between fibroids and preterm birth in a prospective cohort with standardized research ultrasounds for characterizing fibroids in early pregnancy while accounting for the clinical paths that precede preterm birth.

**Methods:**

Participants who were pregnant or planning a pregnancy were recruited from communities in three states between 2000 and 2012. Members of this prospective cohort had a research ultrasound in the first trimester to establish pregnancy dating and to record detailed information about the presence, size, number, and location of fibroids. Baseline information from time of enrollment and a detailed first trimester interview contributed key information about candidate confounders. Birth outcomes, including clinical classification of type of preterm birth (preterm labor, preterm premature rupture of membranes, and medically indicated preterm birth) were cross-validated from participant report, labor and delivery records, and birth certificate data.

**Results:**

Among 4,622 women with singleton pregnancies, 475 had at least one fibroid (10.3%) and 352 pregnancies resulted in preterm birth (7.6%). Prevalence of fibroids was similar for women with preterm and term births (10.2% vs. 10.3%). Fibroids were not associated with increased risk of preterm birth after taking into account confounding (risk ratio adjusted for race/ethnicity and maternal age, 0.88; 95% confidence interval, 0.62–1.24) nor any clinical subtype of preterm birth. No fibroid characteristic or combination of characteristics was associated with risk.

**Conclusions:**

If fibroids increase risk of preterm birth, the effect is substantially smaller than previous estimates. Given lack of effect in a large population of women from the general population, rather than higher risk academic tertiary populations previously most studied, we encourage a reconsideration of the clinical impression that presence of fibroids is a major risk factor for preterm birth.

## Background

Approximately 10% of women have a uterine fibroid detectable by ultrasound in the first trimester [1]. Fibroids are thought to increase preterm birth risk by 50% [2], yet effect estimates range from protective to more than tripling risk [3–18]. The estimated association between specific fibroid characteristics, such as size and number, and preterm birth vary to an even greater extent [4, 14, 19, 20]. Half of studies about fibroids and preterm birth do not account for maternal characteristics that may bias the association, such as maternal age and race, and almost all determine fibroid status via methods prone to misclassification: maternal self-report, insurance codes, medical records, or clinical ultrasounds not intended for fibroid characterization. Preterm birth is the leading cause of neonatal morbidity and mortality in developed countries [21, 22] and a more rigorous evaluation of the relationship between fibroid characteristics and preterm birth is warranted.

Preterm birth is commonly treated as a single outcome. However, multiple etiologic pathways contribute to preterm birth, including spontaneous onset of labor, preterm premature rupture of membranes, and medically indicated delivery for maternal or fetal complications [23, 24]. Each preterm birth subtype has a unique risk profile [25–28]. Studying the association between presence of fibroids and preterm birth subtypes may uncover more insightful risk-relationships [23, 29].

We sought to characterize the association between fibroids and preterm birth in a community-based cohort of women with standardized imaging for fibroid characterization during early pregnancy. We also evaluated the association by clinical subtype of preterm birth with the hypothesis that fibroid status would relate differently to risk of spontaneous versus medically indicated preterm birth.

## Methods

*Right from the Start* is a prospective, community-based pregnancy cohort that recruited women who were pregnant or planning a pregnancy from three states (North Carolina, Tennessee, and Texas) between 2000 and 2012 [30]. Study recruitment materials were distributed through businesses, paid advertising, community groups, and direct mail. Private obstetric and public prenatal care providers also posted flyers and offered brochures about the study. If interested, women were directed to call a toll-free number to be screened for eligibility: aged 18 years and older, trying to become pregnant or pregnant for less than twelve weeks, fluent in English or Spanish, and not using assisted reproductive technologies to conceive. Women planning a pregnancy were provided free pregnancy tests for up to six months and were fully enrolled at first positive pregnancy test. Vanderbilt University’s Institutional Review Board approved study procedures and all participants gave informed consent. Data from this study has also provided evidence about the association between fibroids and first trimester bleeding [31], fibroids and miscarriage [32], fibroids and birthweight [33], and fibroids and C-section risk [34].

Participants completed a baseline interview upon enrollment and a detailed computer-assisted telephone interview in the first trimester. Interviews collected basic demographic information, maternal medical history, reproductive history, and health-related behaviors during early pregnancy.

### Fibroid assessment

Participants had a transvaginal ultrasound for fetal viability assessment, gestational age confirmation, and fibroid characterization. Ultrasounds were performed at a median of 57 days’ gestation (interquartile range [IQR], 48–68 days). Study sonographers with at least five years of obstetric experience followed a detailed protocol for fibroid assessment, which required three sets of caliper measurements for each fibroid’s length, width, and height. Fibroid volume was calculated using the formula for an ellipsoid. Sonographers took multiple images with caliper markings of all fibroids and completed a fibroid map, which indicated fibroid location (cervix, corpus, fundus) and type. Fibroid type was classified as submucosal (distorting or in contact with the uterine cavity without myometrium between fibroid and endometrium), intramural (within the myometrium without distorting the uterine cavity), subserosal (distorting the external contour of the uterus), or pedunculated (located within the uterine cavity and attached by a stalk). Obstetrician investigators masked to pregnancy outcome assessed all images. Gestational age was based on self-reported last menstrual period (LMP) if within seven days of ultrasound predicted gestational age, otherwise ultrasound predicted gestational age was used. Maternal height and weight measured at ultrasound appointment were used to calculate body mass index (BMI).

### Outcome definitions

Participants were followed until pregnancy outcome, which was self-reported and validated by medical and birth certificate records. We defined preterm birth as live birth at less than 37 weeks’ gestation. We categorized preterm birth into three distinct subtypes using hospital records or vital records. Spontaneous preterm labor was defined as onset of spontaneous preterm contractions leading to a preterm birth. Preterm premature rupture of membranes (PPROM) was defined as preterm birth following spontaneous rupture of membranes with subsequent onset of labor. We defined medically indicated preterm birth as labor induction or cesarean birth in the absence of preterm labor or PPROM for maternal or fetal conditions such as pre-eclampsia, fetal growth restriction, or fetal distress. We used the term spontaneous preterm birth to refer to births resulting from either preterm labor or PPROM.

In a secondary analysis, we evaluated preterm birth categories by gestational age. We defined late preterm birth as deliveries occurring for any indication between 34 weeks and 0 days and 36 weeks and 6 days, early preterm birth as deliveries occurring between 28 weeks and 0 days and 33 weeks and 6 days, and very early preterm birth as deliveries occurring prior to 28 weeks.

### Inclusion and exclusion criteria

**Fig. 1:**
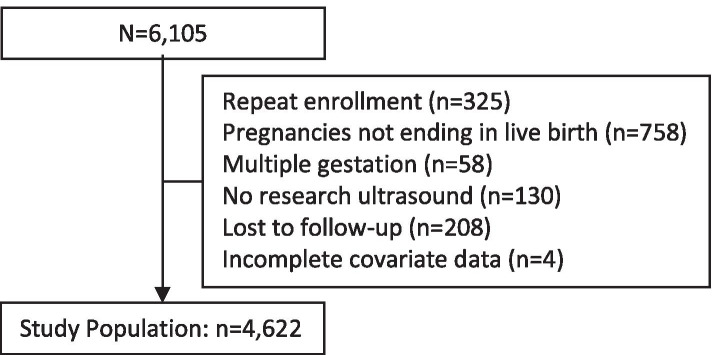
Fibroids and preterm birth study population flow diagram

This analysis was limited to women with a singleton pregnancy resulting in a live birth after 20 weeks’ gestation (a prior analysis of this dataset demonstrated no association between fibroids and spontaneous abortion defined as loss prior to 20 weeks) [32]. If a woman was enrolled for more than one pregnancy, only the first study pregnancy was included in this analysis. We excluded pregnancies without a research ultrasound to confirm fibroid status, and those lacking information about maternal race/ethnicity. Three percent of participants were lost to follow-up (208/6,105). These participants were less likely to have fibroids (3.4% versus 10.3%, chi-squared *p*-value 0.001), were younger (median age 25 versus 29, Wilcoxon rank-sum *p*-value <0.001), and were more likely to be black (35.1% versus 17.4%, chi-squared *p*-value <0.001) compared with participants observed until pregnancy outcome. A total of 4,622 women were included (Fig. 1). All participants enrolled prior to twelve weeks' gestation (median gestational age at enrollment: 46 days’ gestation; IQR, 36-57 days).

### Statistical analysis

We used log-binomial generalized linear regression models to calculate crude and adjusted risk ratios (RRs) and 95% confidence intervals (CIs) associated with overall preterm birth risk for fibroid presence, fibroid number, total fibroid volume (quartiles), largest fibroid volume (quartiles), fibroid type, and location. We also quantified the association between fibroid presence and preterm birth subtypes: spontaneous preterm birth as preterm labor and PPROM and medically indicated preterm birth. We decided *a priori* to include maternal race/ethnicity (white, non-Hispanic; black, non-Hispanic; Hispanic, or other) and age (continuous) in all adjusted models. Other potential confounders (BMI, smoking status, parity, education, and household income) were retained in the final model if their inclusion resulted in a 10% change in the association between fibroids and preterm birth. We tested for effect modification of the relationship between fibroid status and preterm birth by maternal race/ethnicity using the likelihood ratio test. Stratified estimates were reported if the test suggested effect heterogeneity (*p*<0.15).

We did not consider history of preterm birth a confounder in the primary model since fibroid status in prior pregnancies could have impacted prior pregnancy outcome [35, 36]. Instead, we quantified associations with preterm birth and preterm birth subtypes adjusted for history of preterm birth (none, one, two or more) in a sensitivity analysis.

We performed a series of secondary analyses evaluating the association between fibroid presence, number, type, size, and location with late preterm, early preterm, and very early preterm birth using both chi-squared testing and log-binomial generalized linear regression models. We also evaluated the relationship between fibroids with different combinations of characteristics (e.g., multiple large fibroids) and risk of overall preterm birth. We performed all analyses in Stata (Version 14.2, StataCorp, College Station, TX).

## Results

Among 4,622 women, 475 had at least one fibroid on research ultrasound (10.3%) and 352 had a pregnancy ending in preterm birth (7.6%). This population included mostly white or black, non-Hispanic women (71.5% and 17.4%, respectively) from a range of household income and education levels. Forty-eight percent of women were nulliparous and 8.2% reported a prior preterm birth. A notable proportion of women were overweight or obese at pregnancy onset (43.4%) and few smoked (3.5%). Women who were older than 35, black, or obese were more likely to have fibroids (Table 1). Average gestational age at birth for both women with and without fibroids was 39 weeks and 2 days (*p*=0.34, Wilcoxon rank-sum test). Among preterm births, 88% were late preterm (n=309), 29 were early preterm, and 14 were very early preterm.

**Table 1:** Participant characteristics by fibroid status, *Right from the Start*, 2000–2012 (*n* = 4,622)

Characteristic	Fibroids *n* = 475		No Fibroids *n* = 4,147		OR^a^	95% CI
	n	%	n	%		
Maternal Age (years)						
<25	42	8.8	868	20.9	1.00	Referent
25–29	120	25.3	1,531	36.9	1.62	1.13–2.32
30–34	197	41.5	1,294	31.2	3.15	2.23–4.44
≥ 35	116	24.4	454	10.9	5.28	3.64–7.65
Race						
White, non-Hispanic	268	56.4	3,038	73.3	1.00	Referent
Black, non-Hispanic	156	32.8	650	15.7	2.72	2.19–3.37
Hispanic	25	5.3	286	6.9	0.99	0.65–1.52
Other	26	5.5	173	4.2	1.70	1.11–2.62
Parity						
Nulliparous	223	46.9	1,890	45.6	1.00	Referent
1	153	32.3	1,367	33.0	0.95	0.76–1.18
2 +	87	18.3	669	16.1	1.10	0.85–1.43
Missing	12	2.5	221	5.3		
History of SAB						
No	323	68.0	3,097	74.7	1.00	Referent
Yes	140	29.5	829	20.0	1.62	1.31–2.00
Missing	12	2.5	221	5.3		
History of Preterm Birth						
No	419	88.2	3,608	87.0	1.00	Referent
Yes	44	9.3	318	7.7	1.19	0.85–1.66
Missing	12	2.5	221	5.3		
Marital Status						
Married	421	88.6	3,702	89.3	1.00	Referent
Other	54	11.4	445	10.7	1.07	0.79–1.44
BMI^b^						
Underweight	11	2.3	106	2.6	1.18	0.62–2.23
Normal weight	200	42.1	2,266	54.6	1.00	Referent
Overweight	134	28.2	945	22.8	1.61	1.28–2.03
Obese	127	26.7	772	18.6	1.86	1.47–2.36
Missing	3	0.6	58	1.4		
Education						
High school or less	57	12.0	735	17.7	1.00	Referent
Some college	84	17.7	759	18.3	1.43	1.00–2.03
College or more	334	70.3	2,653	64.0	1.62	1.21–2.18
Annual Income ($)						
≤ 40,000	112	23.6	1,184	28.6	1.00	Referent
40,001 to < 80,000	176	37.1	1,468	35.4	1.27	0.99–1.63
≥ 80,000	166	34.9	1,175	28.3	1.49	1.16–1.92
Missing	21	4.4	320	7.7		
Smoking Status^c^						
Never smoker	350	73.7	2,891	69.7	1.00	Referent
Current	34	7.2	488	11.8	0.58	0.40–0.83
Former	83	17.5	576	13.9	1.19	0.92–1.54
Missing	8	1.7	192	4.6		

*Abbreviations*: *OR* Odds ratio, *CI* Confidence interval, *SAB* Spontaneous abortion, *BMI* Body mass index

^a^ Crude odds of exposure given maternal characteristic

^b^ BMI categories from Institute of Medicine guidelines: Underweight < 18.5, Normal weight 18.5–24.99, Overweight 25–29.99, Obese ≥ 30

^c^ Former smoker defined as smoking cessation one month or more before last menstrual period

Prevalence of fibroids in pregnancies ending in preterm and term birth was 10.2% and 10.3%, respectively. Among women with fibroids, 29.3% had more than one. Median total fibroid volume was 4.78 cm^3^ (IQR, 0.97-20.84 cm^3^) and the median volume of the largest fibroid was 4.64 cm^3^ (IQR, 0.83-18.23 cm^3^). Forty-four percent of women with fibroids had at least one intramural fibroid, compared to 42.3% with at least one subserosal and 14.5% with at least one submucosal fibroid. Twenty-one percent of women with fibroids had multiple fibroid types. Fibroid characteristics were similar when comparing women with preterm birth and those who delivered at term (Table 2). Fibroid presence was not associated with overall risk of preterm birth (adjusted RR, 0.88; 95% CI, 0.62–1.24). Neither maternal race nor age modified the association (*p*=0.56 and *p*=0.86, respectively). Fibroid number, volume, type, or location were not associated with preterm birth. We did not identify any combination of fibroid characteristics related to increased risk of preterm birth. When compared to women without fibroids, women with multiple intramural fibroids, intramural fibroids >3 cm in diameter, or multiple fibroids >3 cm in diameter were not at increased risk of preterm birth (analysis not shown).

**Table 2:** Relationship between fibroid characteristics and preterm birth, *Right from the Start*, 2000–2012 (*n* = 4,622)

Fibroid Characteristic	Preterm Births (*n* = 352)		Term Births (*n* = 4,270)		Crude RR	95% CI	Adjusted RR^a^	95% CI
	n	%	n	%				
Fibroid Present								
No	316	89.8	3,831	89.7	1.00	Referent	1.00	Referent
Yes	36	10.2	439	10.3	0.99	0.71–1.39	0.88	0.62–1.24
Fibroids, no.								
0	316	89.8	3,831	89.7	1.00	Referent	1.00	Referent
1	21	6.0	315	7.4	0.82	0.53–1.26	0.76	0.49–1.16
≥ 2	15	4.3	124	2.9	1.42	0.87–2.31	1.14	0.68–1.89
Total Volume^b^								
No fibroids	316	89.8	3,831	89.7	1.00	Referent	1.00	Referent
First quartile	5	1.4	114	2.7	0.55	0.23–1.31	0.55	0.23–1.30
Second quartile	11	3.1	107	2.5	1.22	0.69–2.17	1.12	0.63–1.99
Third quartile	10	2.8	110	2.6	1.09	0.60–2.00	0.95	0.52–1.74
Fourth quartile	10	2.8	108	2.5	1.11	0.61–2.03	0.87	0.47–1.60
Largest Volume^c^								
No fibroids	316	89.8	3,831	89.7	1.00	Referent	1.00	Referent
First quartile	5	1.4	115	2.7	0.55	0.23–1.30	0.55	0.23–1.31
Second quartile	11	3.1	106	2.5	1.23	0.70–2.19	1.10	0.62–1.97
Third quartile	7	2.0	112	2.6	0.77	0.37–1.60	0.66	0.32–1.37
Fourth quartile	13	3.7	106	2.5	1.43	0.85–2.42	1.14	0.67–1.95
Fibroid Type^d^								
Any submucosal	7	2.0	62	1.5	1.33	0.65–2.71	1.09	0.53–2.23
Any intramural	15	4.3	193	4.5	0.95	0.57–1.56	0.81	0.49–1.35
Any subserosal	16	4.5	185	4.3	1.04	0.65–1.69	0.91	0.56–1.49
Any pedunculated	3	0.9	28	0.7	1.27	0.43–3.74	0.99	0.33–2.93
Location ^d^								
Any cervix	6	1.7	64	1.5	1.12	0.52–2.44	1.03	0.47–2.23
Any fundus	16	4.5	172	4.0	1.12	0.69–1.81	0.93	0.57–1.52
Any corpus	21	6.0	242	5.7	1.05	0.69–1.60	0.90	0.58–1.39

*Abbreviations*: *RR* Risk ratio, *CI* Confidence interval

^a^ Adjusted for maternal age and race/ethnicity

^b^ Quartiles for total fibroid volume: < 0.97 cm^3^, 0.97–4.76 cm^3^, 4.77–20.84 cm^3^, > 20.84 cm^3^

^c^ Quartiles for largest fibroid volume: < 0.82 cm^3^, 0.82–4.62 cm^3^, 4.63–18.18 cm^3^, > 18.18 cm^3^

^d^ Columns do not add up to 100% because a participant could contribute to more than one category if she had multiple fibroids, each category is a separate model with women without fibroids as referent group

Preterm birth clinical subtype was known for 60.0% of cases (n=211). Most commonly, preterm birth was secondary to spontaneous preterm labor (n=83, 39.3%), followed by medically indicated delivery for maternal or fetal conditions (n=78, 37.0%), and preterm births after PPROM (n=50, 23.7%). We did not detect an association between fibroid presence and medically indicated preterm birth (adjusted RR, 0.92; 95% CI, 0.43–1.96) or spontaneous preterm birth (adjusted RR, 1.27; 95% CI, 0.76– 2.11; Table 3). Findings did not change when adjusted for prior preterm birth.

**Table 3:** Relationship between fibroid status and preterm birth by subtype, *Right from the Start*, 2000–2012 (n = 4,622)

Outcome	Preterm	Unadjusted RR	95% CI	Adjusted RR^a^	95% CI	Adjusted RR, Secondary^b^	95% CI
All preterm births	352	0.99	0.71–1.39	0.88	0.62–1.24	0.93	0.66–1.30
Spontaneous	133	1.35	0.83–2.20	1.27	0.76–2.11	1.29	0.77–2.17
Preterm labor	83	1.19	0.62–2.29	1.31	0.66–2.59	1.43	0.73–2.82
PPROM	50	1.65	0.78–3.49	1.18	0.53–2.62	1.12	0.48–2.58
Medically indicated	78	1.00	0.48–2.06	0.92	0.43–1.96	1.02	0.48–2.18

^a^ All analyses are from separate models comparing preterm birth (or preterm birth subtype) to 4,270 term births, adjusted for maternal age and race/ethnicity

^b^ Secondary analysis adjusted for maternal age, race/ethnicity, and history of preterm birth

Fibroid presence or size was not associated independently with late preterm, early preterm, or very early preterm birth. However, the number of women with deliveries prior to 34 weeks of gestation was small, and therefore this cohort is inadequately powered to assess risk for early and very early preterm births.

## Discussion

In this prospective, community-based pregnancy cohort with standardized imaging for fibroid characterization in the first trimester, we did not find evidence that fibroid presence, size, location, or number influences risk of preterm birth. Fibroids were not associated with any clinical subtype of preterm birth. If fibroids increase preterm birth risk, the effect is notably more modest than previous literature indicates.

Given the null association between fibroids and preterm birth, let us consider the confidence we have in these results. Participants had a research ultrasound to determine fibroid presence, size, location, and type in the first trimester. Imaging was performed by experienced clinical sonographers using a detailed protocol to systematically characterize fibroids. This is an improvement over studies depending on maternal self-report, which fails to capture up to 80% of fibroids [37, 38], or retrospective ultrasound databases, which rely on imaging not meant to uniformly detect or characterize fibroids, and are therefore prone to misclassification. More rigorous methods for fibroid classification in this study better capture exposure and account for the higher prevalence of fibroids observed in this cohort compared with other studies.

We used community-based recruitment methods to enroll a cohort more representative of the general population than clinic-based studies [39]; though we acknowledge that those who volunteer to participate in a study of pregnancy health may be more health-conscious than those who do not. Additionally, we recruited women prior to conception or in the first trimester to ensure standardized fibroid assessment in early pregnancy. While this results in more rigorous exposure classification, it also necessitates that participants identified pregnancy early. These two factors may lead to a lower risk cohort than the general population.

We excluded women who used reproductive technologies to conceive from this analysis. The association between fibroids and preterm birth may be underestimated if fibroid characteristics linked to infertility also drive risk of preterm birth. Nonetheless, fibroid presence, size, and type were not associated with time to pregnancy in this cohort [40]. We did not find that maternal age or race modified the association between fibroids and preterm birth. However, further questions about risk attributable to fibroids in the setting of other factors such as prior myomectomy, prior C-sections, or the use of assisted reproductive technologies should be explored.

In most studies about fibroids and preterm birth [3–8, 10–18], spontaneous and medically indicated preterm birth are treated as the same outcome. Proposed biological mechanisms for risk associated with fibroids are architectural in nature (fibroids prevent proper placentation, impair distensibility of the uterus, cause uterine irritability and preterm contractions, or lead to intrauterine crowding) and are more in line with how fibroids may contribute to spontaneous preterm birth. We endeavored to determine if distinct relationships exist between fibroid status and preterm birth subtypes. Though women were followed prospectively in this cohort until pregnancy outcome, the effort to determine the subtype of preterm birth (spontaneous versus medically indicated) was done retrospectively. Information concerning the birth was insufficient to confidently classify subtype in 40.0% of cases and precision of subtype-specific estimates was limited. Consistent with a prospective study about predictors of medically indicated preterm birth, we did not find evidence fibroids are associated with increased risk of this subtype after adjusting for maternal age and race [25]. However, Meis et al. study relied on ultrasound reports not standardized to assess fibroid status, resulting in possible misclassification of fibroid status as suggested by the low prevalence of detected fibroids in the study population (1.5%) [9]. Due to the small number of cases for individual preterm birth subtypes, we could not assess how fibroid number, size, location, and type related to specific subtypes. Future studies about the relationship between fibroid characteristics and specific preterm birth subtypes are warranted.

## Conclusions

We did not find evidence fibroids contribute to preterm birth risk in this prospective study of more than four thousand women, nor did we detect an association between fibroids and any clinical preterm birth subtype. To abate undue anxiety among expectant mothers, we encourage a reassessment of classifying presence of fibroids as a risk factor for preterm birth among women with normal fertility.

## Data Availability

The datasets and materials used in the study are available from the corresponding author (Dr. Hartmann email address) via concept proposal request to the study senior investigators. Additional information about requesting *Right from the Start* data can be found at https://rightfromthestartstudy.org/collaborate/.

## Abbreviations

BMI: Body mass index
CI: Confidence intervals
IQR: Interquartile range
LMP: Last menstrual period
OR: Odds ratio
RR: Risk ratio
PPROM: Preterm premature rupture of membranes
SAB: Spontaneous abortion
